# Leprosy reactions: The predictive value of *Mycobacterium leprae*-specific serology evaluated in a Brazilian cohort of leprosy patients (U-MDT/CT-BR)

**DOI:** 10.1371/journal.pntd.0005396

**Published:** 2017-02-21

**Authors:** Emerith Mayra Hungria, Samira Bührer-Sékula, Regiane Morillas de Oliveira, Lúcio Cartaxo Aderaldo, Araci de Andrade Pontes, Rossilene Cruz, Heitor de Sá Gonçalves, Maria Lúcia Fernandes Penna, Gerson Oliveira Penna, Mariane Martins de Araújo Stefani

**Affiliations:** 1 Instituto de Patologia Tropical e Saúde Pública, Universidade Federal de Goiás, Goiânia, GO, Brasil; 2 Centro de Dermatologia Dona Libânia, Fortaleza, Ceará, Brasil; 3 Fundação de Dermatologia Tropical e Venereologia Alfredo da Matta, Manaus, Amazonas, Brasil; 4 Departamento de Epidemiologia e Bioestatística, Universidade Federal Fluminense, Rio de Janeiro, Rio de Janeiro, Brasil; 5 Núcleo de Medicina Tropical, Universidade de Brasília, Brasília, DF, Brasil; Fondation Raoul Follereau, FRANCE

## Abstract

**Background:**

Leprosy reactions, reversal reactions/RR and erythema nodosum leprosum/ENL, can cause irreversible nerve damage, handicaps and deformities. The study of *Mycobacterium leprae*-specific serologic responses at diagnosis in the cohort of patients enrolled at the *Clinical Trial for Uniform Multidrug Therapy Regimen for Leprosy Patients in Brazil/U-MDT/CT-BR* is suitable to evaluate its prognostic value for the development of reactions.

**Methodology:**

IgM and IgG antibody responses to PGL-I, LID-1, ND-O-LID were evaluated by ELISA in 452 reaction-free leprosy patients at diagnosis, enrolled and monitored for the development of leprosy reactions during a total person-time of 780,930 person-days, i.e. 2139.5 person-years, with a maximum of 6.66 years follow-up time.

**Principal findings:**

Among these patients, 36% (160/452) developed reactions during follow-up: 26% (119/452) RR and 10% (41/452) had ENL. At baseline higher anti-PGL-I, anti-LID-1 and anti-ND-O-LID seropositivity rates were seen in patients who developed ENL and RR compared to reaction-free patients (p<0.0001). Seroreactivity in reactional and reaction-free patients was stratified by bacilloscopic index/BI categories. Among BI negative patients, higher anti-PGL-I levels were seen in RR compared to reaction-free patients (p = 0.014). In patients with 0<BI<3, (36 RR, 36 reaction-free), higher antibody levels to PGL-I (p = 0.014) and to LID-1 (p = 0.035) were seen in RR while difference in anti-ND-O-LID positivity was borderline (p = 0.052). Patients with BI≥3 that developed ENL had higher levels of anti-LID-1 antibodies (p = 0.028) compared to reaction-free patients. Anti-PGL-I serology had a limited predictive value for RR according to receiver operating curve/ROC analyses (area-under-the-curve/AUC = 0.7). Anti LID-1 serology at baseline showed the best performance to predict ENL (AUC 0.85).

**Conclusions:**

Overall, detection of anti-PGL-I, anti-LID-1 and anti-ND-O-LID antibodies at diagnosis, showed low sensitivity and specificity for RR prediction, indicating low applicability of serological tests for RR prognosis. On the other hand, anti-LID-1 serology at diagnosis has shown prognostic value for ENL development in BI positive patients.

**Trial Registration:**

ClinicalTrials.gov NCT00669643

## Author summary

Leprosy is a debilitating dermato-neurologic disease caused by *Mycobacterium leprae*. One of the main difficulties in the clinical management of leprosy patients is the development of leprosy reactions which are immune inflammatory episodes that can cause irreversible handicaps, incapacities and deformities. There are two major types of leprosy reactions: reversal reaction (RR) and erythema nodosum leprosum (ENL). Currently, there is no laboratory test able to predict the emergence of leprosy reactions among recently diagnosed patients. In order to investigate laboratory markers for the occurrence of leprosy reactions, we investigated the prognostic value of serologic responses to *M*. *leprae* antigens (PGL-I, LID-1, ND-O-LID) in 452 leprosy patients enrolled at the *Clinical Trial for Uniform Multidrug Therapy Regimen for Leprosy Patients in Brazil/U-MDT/CT-BR*. At diagnosis higher anti-PGL-I, anti-LID-1 and anti-ND-O-LID seropositivity rates were seen in patients who developed ENL and RR compared to reaction-free patients. The anti-PGL-I serology at diagnosis show low sensitivity to predict RR and anti-LID-1 serology at diagnosis has shown prognostic value for ENL development.

## Introduction

Leprosy is a complex dermato-neurologic disease caused by *Mycobacterium leprae* that presents a wide spectrum of clinical manifestations characterized by distinct bacteriologic, immunologic and histopathologic features [1]. On one pole, tuberculoid leprosy (TT) is characterized by few skin lesions, low or absent bacilloscopic index (BI), strong *M*. *leprae*-specific Th1-type cell-mediated immunity (CMI) and low or absent specific antibodies. On the other extreme of the spectrum, lepromatous leprosy (LL) is characterized by multiple disseminated skin lesions, high BI, Th2-type immunity with vigorous antibody production and low or absent *M*. *leprae*-specific CMI. Additionally, immunologically unstable borderline forms (borderline tuberculoid/BT, borderline borderline/BB and borderline lepromatous/BL) lie in the middle of the spectrum combining features of both poles [2].

Leprosy treatment, known as multidrug therapy (MDT) is based on different combinations of antibiotics for paucibacillary (PB) and multibacillary (MB) leprosy, classified according to the number of skin lesions (< = 5: PB leprosy; >5: MB leprosy). MB leprosy patients are prescribed with 12 monthly-supervised doses of rifampicin, dapsone and clofazimine plus self-administered daily doses of dapsone and clofazimine. PB leprosy patients are treated with six monthly-supervised doses of rifampicin and dapsone plus self-administered daily doses of dapsone [3]. In 2007, an open-label, randomized clinical trial was designed and conducted to compare the regular MDT (R-MDT) proposed by WHO and a uniform MDT regimen (U-MDT) consisting of six doses of rifampicin, dapsone and clofazimine for PB and MB leprosy patients. Clinical monitoring is still under way in Brazil regarding mainly the development of reactions and relapses *(Clinical Trial for Uniform Multidrug Therapy Regimen for Leprosy Patients in Brazil*, *U-MDT/CT-BR)* [4–8].

One of the main difficulties in the clinical management of leprosy patients is the development of leprosy reactions that can occur anytime during the chronic course of the disease: before diagnosis, during treatment and even years after treatment release [2, 9, 10]. Leprosy reactions represent immunologically mediated episodes of acute inflammation that if not diagnosed and treated promptly can cause irreversible impairment of nerve function and permanent incapacities [11]. There are two major types of leprosy reactions: type 1 reaction (T1R) or reversal reaction (RR) which is associated with Th1-type immunity and type 2 reaction (T2R) represented mainly by erythema nodosum leprosum (ENL) which is related to Th2-type immune responses [9, 12]. Currently, there is no laboratory test able to predict the emergence of leprosy reactions among recently diagnosed patients.

Leprosy serology, comprises the well known detection of IgM antibodies against the phenolic glycolipid I (PGL-I), a *M*. *leprae* specific cell-wall antigen. Since the PGL-I identification, several studies have been extensively performed to understand the use of this antigen in diagnostic tests and the immune response in leprosy, but there are still many knowledge gaps to be filled [13]. More recent IgG based tests to newly *M*. *leprae*-recombinant protein antigens have been described [14, 15]. In leprosy, the seropositivity of IgM and IgG tests reflects the patients’ bacillary load with low positivity rates in PB patients and high positivity in MB patients [16, 17]. After the decodification of *M*. *leprae* genome, over 200 new recombinant proteins have been screened in serology and cell mediated tests aiming the development of new diagnostic tests for leprosy [15, 18–23]. Results from serological screenings in different endemic areas in the world have highlighted the significant reactivity of ML0405 and ML2331 proteins, which were later fused and named LID-1 antigen (*Leprosy IDRI Diagnostic-1*). LID-1 was shown to retain the immunogenicity of the original proteins with high positivity rates reported in MB leprosy patients from different endemic sites [18, 24]. PGL-I and LID-1 antigens represent important specific antigens for leprosy serology and their association was suggested to improve the sensitivity of serology for MB leprosy [25]. In this regard, a single fusion complex (ND-O-LID) resulting from the conjugation of the synthetic mimetic disaccharide of PGL-I (ND-O) and LID-1 was constructed and shown to detect most MB leprosy by both a lateral flow platform and by ELISA [26, 27].

Our results from a previous semi-quantitative serological study with the entire cohort of *U-MDT/CT-BR* showed that results of rapid lateral flow test (ML Flow) to detect IgM antibodies to PGL-I antigen at diagnosis had low sensitivity and specificity to predict the development of leprosy reactions during follow-up [28]. This previous results obtained using the ML Flow, prompted us to investigate the predictive value for leprosy reactions of the quantitative serology to new *M*. *leprae* protein antigens as LID-1 and ND-O-LID compared to the well known anti PGL-I serology. This investigation used a robust sera bank of the *U-MDT/CT-BR*, a well characterized cohort of leprosy patients monitored for more than 6 years regarding the development of leprosy reactions and relapses. [4, 6–8, 29].

## Materials and methods

### Study population

Our study group included 452 out of 753 patients who were enrolled at the *U-MDT/CT-BR* (March 2007-September 2013) in two highly endemic areas for leprosy in Brazil (Fortaleza, Ceará, northeast; Manaus, Amazonas, north region). So far, patients have been followed for a total person-time of 780,930 person-days, i.e. 2139.5 person-years, with a maximum of 6.66 years follow-up time [8]. At enrollment, all leprosy patients had complete dermato-neurological evaluation by clinicians with vast expertise in leprosy diagnosis. The following laboratory tests were performed at diagnosis: ML Flow rapid test, slit skin smear, histopathology of biopsies from leprosy skin lesions. For research purposes, patients were categorized according to a modified Ridley-Jopling (R&J) classification system considering clinical features, histopathology of skin lesions and the slit skin smear bacterial index (BI). Mitsuda tests and BI of the skin lesions were not performed.

In this case-control study, we have compared serology results at diagnosis from patients that developed reactions during follow-up (RR and ENL, without other complications) and patients that remained reaction-free during entire monitoring. From the original group of 753 patients, exclusions (n = 301) were due to: unavailability of serum sample at diagnosis (n = 46); reaction at diagnosis (RR = 16), reaction associated or not with neuritis (n = 184) or other clinical manifestations such as orchitis, arthritis and lymphadenopathy (n = 55).

The socio-demographic, clinical and laboratory characteristics of our study group are similar to the previously described characteristics of the entire group [30] (**S1 Table**). In our study-group, 26% patients developed RR (119/452), 10% had ENL (41/452) while 64% remained reaction-free (292/452). The majority of patients was male, the age of patients who developed RR was similar compared to reaction-free patients (median age of 43 and 41 years respectively, p = 0.44), while patients who developed ENL were younger (median age = 35 years) compared to reaction-free and RR patients (p = 0.003 and p = 0.001, respectively). Most reactional patients was classified as BL leprosy (62%, 99/160), whereas most reaction-free patients was classified as BT leprosy (60%, 176/292).

The baseline serological profiles to PGL-I, LID-1 and ND-O-LID antigens in different Ridley & Jopling groups is in agreement with previous reports [15, 18–23]. Higher levels of IgM anti-PGL-I and IgG anti-LID-1 antibodies were detected in MB leprosy (BB, BL, LL) compared to PB leprosy (TT, BT) (p<0.0001). Higher positivity of IgM and IgG responses to ND-O-LID antigens was found in BL and LL patients compared to TT, BT groups (p<0.0001) (**S1 Fig**).

### Detection of IgM antibodies to PGL-I

Serum IgM antibodies to PGL-I were detected by ELISA performed as described previously [31]. PolySorp 96-well plates (Nunc, Roskilde, Denmark) were coated with 0.01 μg/mL of the semi-synthetic analogue of PGL-I (NT-P-BSA; batch: Nara XVI-61, Dr Fujiwara, Japan), or BSA and blocked with PBS-Tween containing 1% BSA. Serum samples diluted 1/300 in PBS-T containing 10% normal goat serum/NGS (Sigma-Aldrich, St. Louis, USA) were incubate and washing, horse radish peroxidase/HRP-conjugated anti-human IgM (Immuno Chemicals, St. Louis, Missouri, USA) was added. After incubation and washing, peroxidase color substrate (TMB, Sigma Aldrich, St. Louis, USA), was added and the reaction was quenched by the addition of 2.5 N H_2_SO_4_, when the optical density/OD at 450 nm from reference serum reached an OD value of 0.6 (Bio-Rad microplate reader, Life Science, Hercules, CA, USA). The final OD was calculated by subtracting the OD values of BSA coated wells from OD values of NT-P-BSA wells. The cut-off was defined as OD > 0.25 as previously described [16].

### Detection of IgG antibodies to recombinant proteins

Serum IgG antibodies to LID-1 (batch: 14 November 2011, Dr Duthie, USA) and the single fusion complex (ND-O-LID- batch: 17 August 2012, Dr Duthie, USA) were detected by ELISA. Polysorp 96 well plates (Corning Costar, NY, USA) were coated with 1μg/mL LID-1 or with 0.25 μg/mL ND-O-LID. Blocking was performed with PBS-T 1% BSA. Serum samples diluted 1/200 in PBS-T-10% NGS were added in duplicate and incubated for 2 hours at room temperature. Plates were washed and incubated for 1 hour with HRP-conjugated anti-human IgG (Southerm Biotech, Birmingham, AL) for anti LID-1 serology and anti-human IgG (Southerm Biotech, Birmingham, AL) plus anti-human IgM (Immuno Chemicals, St. Louis, Missouri, USA) for anti- ND-O-LID serology. After washing, reactions were developed with peroxidase color substrate (KPL, Gaithersburg, MD, USA) and quenched by the addition of 1 N H_2_SO_4_. The optical density was determined (Bio-Rad microplate reader, Life Science, Hercules, CA, USA) at 450 nm. For anti LID-1 serology the cut-off was calculated to be 2 times the standard deviation of the OD of sera from healthy endemic controls (EC), such that samples with OD > 0.3 were considered positive [18]. As previously described, the anti-ND-O–LID serology threshold for positive responses was considered OD > 0.923 [20]. The results of serologic tests were expressed as the mean OD of duplicates.

### Statistical analyses

The baseline serologic profiles of PB and MB patients enrolled in the *U-MDT/CT-BR* were analyzed according to the clinical outcomes reported during clinical follow-up, which included reactional (RR and ENL) and reaction-free patients. In this study group, the frequency of leprosy reactions was similar in the first year after diagnosis compared to subsequent monitoring (p>0.05), therefore, the consolidated data analysis, considered the predictive value of baseline serology for the development of leprosy reactions observed any time during the entire monitoring up to 6.66 years after MDT. Serological results were analyzed based on the patients´ bacteriological index (BI) which was stratified into 3 categories: negative BI, BI higher than zero and lower than 3 (0> BI < 3) and BI equal or higher than 3 (BI≥ 3) as previously reported [8].

Statistical significance was assessed using the Kruskal-Wallis one-way analysis of variance for comparison of multiple groups and by the Mann-Whitney *U* test for comparison between two groups. *Chi* square (χ2) was used to compare positivity rates. Receiver Operating Curves (ROC) were determined with 95% confidence interval (CI) (GraphPad Prism, version 5) and results were considered statistically significant when p values <0.05 were obtained.

### Ethical considerations

The UMDT/CT-BR trial was performed considering international (Helsinki) and Brazilian research regulations involving human beings and approvals were obtained from all the regional research ethical committees involved and from the National Committee for Ethics in Research (CONEP) of the National Health Council/ Ministry of Health, Brazil (protocol # 001/06). Additionally, approved by the human and animal research ethics committee from Federal University of Goiás (CEMHA/HC/UFG#166/2011). Written informed consent was obtained from all patients prior to inclusion in the study. For patients under 18 years old, written parental consent was obtained. Data confidentiality was strictly guaranteed and all patients were informed they were free to leave the study and opt for the regular MDT treatment (ClinicalTrials.gov identifier: NCT00669643).

## Results

### Higher antibody levels and seropositivity at diagnosis in reactional *versus* reaction-free patients

**Fig 1:**
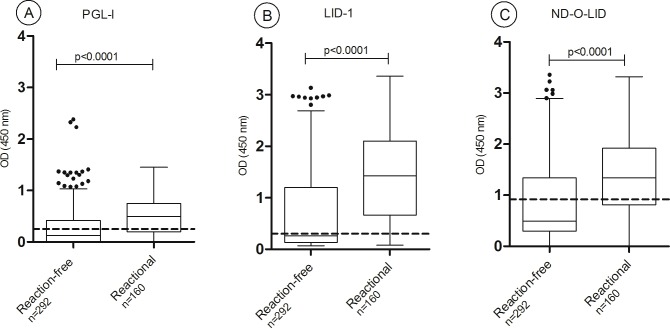
Baseline antibody responses to PGL-I (A), LID-1 (B) and ND-O-LID (C) in reaction-free (n = 292) and reactional leprosy patients (RR+ENL, n = 160). The box shows the interval between the first and the third quartiles, the middle line represents the median. The *p* value refers to differences in OD medians. The traced horizontal line is the cut-off: PGL-I OD>0.25; LID-1 OD>0.3; ND-O-LID OD>0.923. The numbers above each box represent the positivity rate and the points above each box are outlier results. OD = optical density.

Higher IgM anti PGL-I seropositivity was observed in reactional patients compared to reaction-free ones (73%, *versus* 39%; p<0.0001). Similarly, the median ODs were higher among reactional compared to reaction-free patients (0.498 *versus* 0.128, respectively; p<0.0001). Regarding anti-LID-1 and anti-ND-O-LID serology in reactional *versus* reaction-free patients, both the positivity rates and the antibody levels, measured by median OD, were higher in reactional patients: IgG antibodies to LID-1 (84% *vs* 46% positivity, median ODs = 1.43 and 0.261 respectively, p>0.0001), IgM and IgG antibody levels to ND-O-LID antigens (67% *vs* 32% positivity, median ODs = 1.34 and 0.493 respectively, p<0.0001) (Fig 1). However, 2–6% reaction-free patients had outliers serological results for the three antigens tested.

We have investigated if the time elapsed between blood intake and the development of reaction has had any effect on serology as ongoing “subclinical reactions” could have impacted the antibody titers of patients that manifested reactions within the first year of monitoring (n = 68). For these analyses we have separated patients into three groups according to the time between these two events (diagnosis/intake and development of reactions): 90 days, 90–180 days and 180–365 days. These analyses showed that regardless of the time in which reactions occurred after the first year of leprosy diagnosis, patients that further developed reactions showed highest the O.D medians for all antigens evaluated compared to reaction-free patients (S2 Fig).

### Higher antibody levels to PGL-I, LID-1 and ND-O-LID antigens in ENL and RR *versus* reaction-free patients

**Fig 2:**
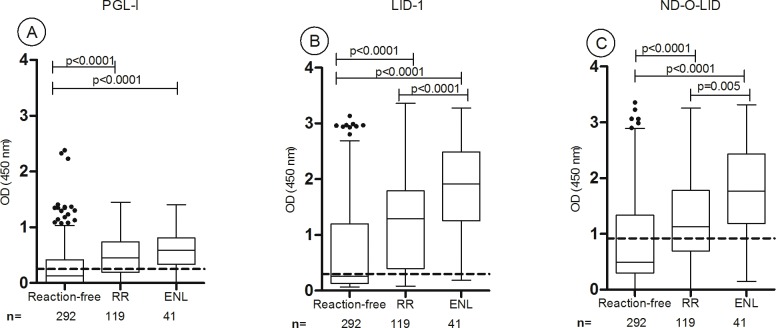
Baseline antibody responses to PGL-I (A), LID-1 (B) and ND-O-LID (C) among reaction-free (n = 292), leprosy patients that developed RR (n = 119) and leprosy patients that developed ENL (n = 41). The box shows the interval between the first and the third quartiles, the middle line represents the median. The *p* value refers to differences in OD medians. The traced horizontal line is the cut-off: PGL-I OD>0.25; LID-1 OD>0.3; ND-O-LID OD>0.923. The numbers above each box represent the positivity rate and the points above each box are outliers results. OD = optical density; RR: reversal reaction; ENL: erythema nodosum leprosum.

At diagnosis higher anti-PGL-I positivity rates were seen in patients who developed both ENL (78%, 32/41) and RR (76%, 90/119) (p = 0.376) compared to reaction-free patients (39%, 113/292; p<0.0001). Similarly, anti-LID-1 positivity was higher in reactional (ENL: 95%, 39/41; RR: 80%, 95/119) compared to reaction-free patients (46%, 134/292) (p>0.0001). Regarding LID-1 serology at baseline, patients that developed ENL during follow-up had higher positivity than patients that manifested RR (p = 0.01). Serological responses to ND-O-LID were higher in ENL patients (88%, 36/41) compared to RR patients (60%, 71/119; p = 0.0004) and reaction-free ones (32%, 93/292; p<0.0001) (Fig 2).

For all three *M*. *leprae* antigens tested, the ELISA results showed a gradual increase in the median ODs. The lowest median OD was seen in reaction-free patients, increasing in RR patients and the highest values were seen in ENL patients. For PGL-I serology, the median OD among reaction-free patients was 0.128 (range 0–2.38) while among patients that developed RR and ENL the median ODs were 0.45 (range 0–1.45) and 0.58 (range 0–1.41), respectively (reaction-free *versus* RR and reaction-free *versus* ENL, p<0.0001). For LID-1 serology, reaction-free patients presented a median OD of 0.26 (range 0.07–3.13) while higher antibody levels were seen in patients that developed RR (median OD = 1.29; range 0.08–3.36) and ENL (median OD = 1.9; range 0.18–3.27). Differences in antibody levels (measured by the median OD) were statistically significant when comparing reactional patients (RR or ENL) with reaction-free group (p<0.0001) and when comparing RR and ENL groups (RR *versus* ENL, p<0.0001) (Fig 2). The IgG and IgM antibody levels to ND-O-LID were also higher among reactional patients (ENL, median OD = 1.77, range 1.47–3.32 and RR, median OD = 1.11, range 0–3.25) when compared to reaction-free patients (median OD = 0.49; range 0–3.36, p<0.0001). Higher antibody levels were detected in ENL patients compared to RR (p = 0.005).

### Antibody levels analyzed according to the bacteriological index at diagnosis in patients that developed reactions during follow-up and in reaction-free leprosy patients

As we observed a gradual increase in antibody levels from reaction–free to RR and ENL patients and since in leprosy, antibody responses are positively correlated with patients’ BI, we have analyzed the impact of BI in serology from reactional *versus* reaction-free patients. The analysis of BI and serological levels to *M*. *leprae*-specific antigens for both reactional and reaction-free groups clearly indicated that for RR and reaction-free patients, serological levels were associated with BI (**S3 Fig**). However, for patients that further developed ENL and who had high BIs (>3), despite inter-individual serologic variability, antibody levels had not correlation with BI.

Therefore we have analysed, serologic responses to PGL-I, LID-1 and ND-O-LID in reactional and reaction free patients stratified according to the bacterial index range: BI = 0, 0<BI<3 and BI≥3.

### In BI negative patients, RR had higher anti-PGL-I levels compared to reaction-free patients

**Fig 3:**
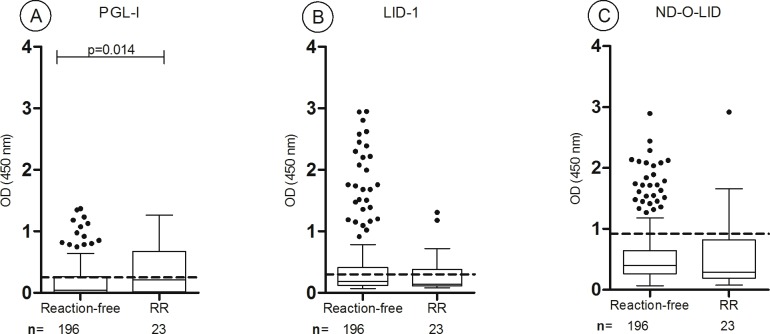
Group 1- BI negative patients: Antibody responses to PGL-I (A), LID-1 (B) and ND-O-LID (C) antigens in reaction-free patients (n = 196) and patients that developed RR (n = 23). The box shows the interval between the first and the third quartiles, the middle line represents the median. The *p* value refers to differences in medians of OD. The traced horizontal line is the cut-off value: PGL-I OD>0.25; LID-1: OD>0.3; ND-O-LID: OD>0.923. The numbers above each box represent the positivity rate and the points above each box are outlier results. OD = optical density; RR: reversal reaction.

The BI negative patients were mostly reaction-free (n = 196, 89%) while 23 developed RR during clinical monitoring and no case of ENL was reported in this group. Serology of reaction-free BI negative leprosy patients (33 TT, 161 BT, 2 BB) compared to RR patients (2 TT, 20 BT, 1 BL) shower higher anti-PGL-I positivity rate in RR (27% *versus* 49%, respectively, p = 0.02). The difference in anti- PGL-I antibody levels (median ODs) between reaction-free and RR patients was also statistically significant (median ODs of 0.04 and 0.213 respectively, p = 0.014) (Fig 3). Anti-PGL-I positivity of BI negative patients that developed RR was higher compared to reaction-free ones. However, among reaction-free patients, 10–15% had high outlier serologic results to PGL-I, LID-1, ND-O-LID indicating an important overlap of serologic responses from reaction-free patients and patients who developed RR. Extended clinical monitoring of these patients reaction-free patients with high seropositivity for 2 further years (until December 2015) did not reveal the development of reactions nor relapse in any of these patients.

### In patients with BI 0<BI<3, RR patients had higher levels of anti-PGL-I and anti-LID-1 antibodies compared to reaction-free patients

**Fig 4:**
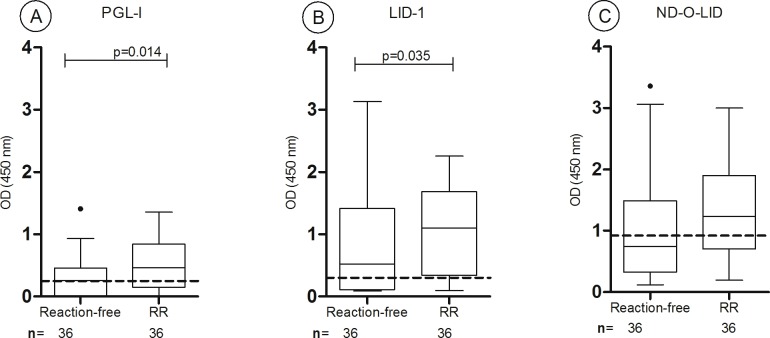
Group 2- Patients with 0<BI<3 (n = 72): antibody responses to PGL-I (A), LID-1 (B) and ND-O-LID (C) among reaction-free patients (n = 36) and patients that developed RR (n = 36). The box shows the interval between the first and the third quartiles, the middle line represents the median. The *p* value refers to differences in OD medians. The traced horizontal line is the cut-off: PGL-I OD>0.25; LID-1: OD>0.3; ND-O-LID: OD>0.923. The numbers above each box represent the positivity rate and the points above each box are outliers results. OD = optical density; RR: reversal reaction.

This group of patients was composed by equal numbers of reaction-free patients (n = 36; 13 BT, 7 BB, 15 BL, 1 LL) and patients that developed RR (n = 36; 8 BT, 3 BB, 25 BL). Seropositivity to PGL-I and LID-1 antigens was higher in reactional patients compared to reaction-free patients, 72% and 78% of patients who developed RR during follow-up were positive at diagnosis while among reaction-free patients, 50% and 53% were positive (p = 0.026 and p = 0.012 respectively). Anti-ND-O-LID positivity rates were similar in reaction-free and RR groups (p>0.05) (Fig 4). For anti-PGL-I and anti-LID-1 serology, patients who developed RR had higher median ODs compared to reaction-free ones (PGL-I, median ODs = 0.46 and 0.25; p = 0.014 and LID-1, median ODs = 1.1 and 0.52; p = 0.035). For anti-ND-O-LID serology, the difference in antibody levels between groups was marginal (p = 0.052) (Fig 4).

### In patients with BI≥3, RR patients had higher anti-LID-1 seropositivity; ENL patients had higher levels of anti-LID-1 antibodies and anti-ND-O-LID seropositivity

**Fig 5:**
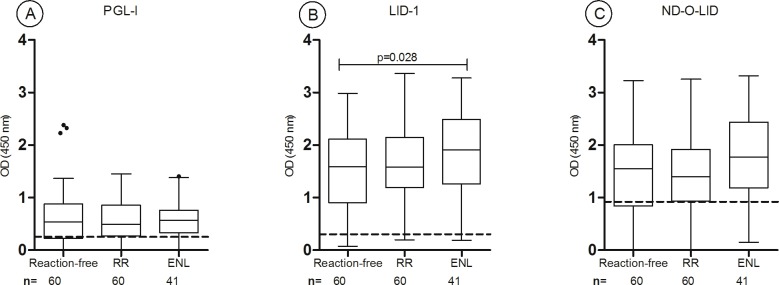
Group 3- Patients with BI≥3 (n = 161): Baseline antibody responses to different *M*. *leprae* antigens. Seropositivity to PGL-I (A), LID-1 (B) and ND-O-LID (C) antigens in patients that developed RR (n = 60), ENL (n = 41) and reaction-free (n = 60) patients. The box shows the interval between the first and the third quartiles, the middle line represents the median. The *p* value refers to differences in OD median. The traced horizontal line is the cut-off: PGL-I OD>0.25; LID-1: OD>0.3; ND-O-LID: OD>0.923. The numbers above each box represent the positivity rate and the points above each box are outliers results. OD = optical density; RR: reversal reaction; ENL: erythema nodosum leprosum.

This group of patients was composed by reaction-free patients (n = 60; 34 LL, 2 BT, 24 BL) and patients that developed either RR (n = 60; all BL) or ENL (n = 41; 28 LL, 13 BL). Although RR patients presented higher seropositivity rate for all tested antigens, this difference was only significant for anti-LID-1 serology (p = 0.01). However, reaction-free and RR patients had similar medians of ODs for all tested antigens (p>0.05). Anti-ND-O-LID baseline positivity rates were higher in ENL patients compared to reaction-free patients (88% *versus* 72% respectively, p = 0.03). Also, for LID-1 serology, patients that developed ENL had higher median OD than reaction-free patients (OD median = 1.91 and 1.58, respectively; p = 0.028) (Fig 5A, 5B and 5C).

### At baseline: Can anti-PGL-I serology predict the development of RR? Can anti-PGL-I, anti-LID-1 and anti-ND-O-LID responses predict the development of ENL?

The accuracy of serology at diagnosis, using PGL-I, LID-1 and ND-O-LID antigens, to predict the development of leprosy reactions during follow-up was analyzed by ROC curve. According to this analysis, a test that gives an area under the curve (AUC) above 0.7 is considered satisfactory [32].

**Fig 6:**
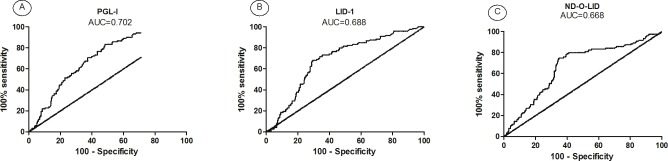
Receiver Operating Curve (ROC) for serology to (A) PGL-I, (B) LID-1 and (C) ND-O-LID antigens in leprosy patients who developed RR during follow-up and reaction-free.

For RR, comparison of the antibody responses in RR *versus* reaction-free patients, only anti PGL-I serology presented AUC above 0.7 (Fig 6) but all antigens presented similar AUC. Establishing a specificity of 80% (95% CI: 75–84%) the sensitivity is 44% (95% CI: 35–54%) for a *cut-off* OD > 0.521. Sensitivity, specificity and the AUC of *M*. *leprae*-specific serology is detailed in **S2 Table**.

**Fig 7:**
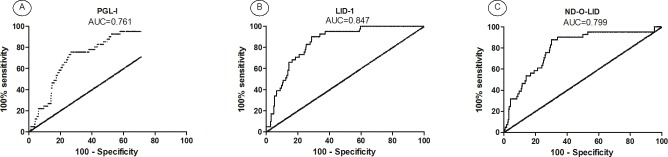
Receiver Operating Curve (ROC) for (A) PGL-I, (B) LID-1 and (C) ND-O-LID antigens in leprosy patients who developed ENL during follow-up and reaction-free.

Regarding ENL during follow-up, all three serologic tests showed acceptable results. For anti PGL-I and anti ND-O-LID serology, establishing a specificity of 80% (95% CI: 75–84%) the sensitivity is 58% (95% CI: 42–73%) for a *cut-off* O.D > 0.520 and 1.527, respectively. Anti LID-1 serology at baseline showed the best performance to predict ENL (AUC = 0.847). Setting a specificity of 80% (95% CI: 75–84%) and sensitivity of 71% (95% CI: 55–84%) the *cut-off* point was O.D > 1.5 (Fig 7 and S2 Table).

## Discussion

Leprosy reactions are immune inflammatory episodes that can cause irreversible handicaps, incapacity and deformities and no prognostic marker is currently available [9]. In the current study, the use of quantitative *M*. *leprae* specific ELISAs with PGL-I, LID-1 and NDO-LID antigens, that have high sensitivity for multibacillary patients, have shown the potential application of anti-LID-1 serology at diagnosis for ENL prediction. This finding is in accordance with previous reports indicating that ENL occurs in multibacillary patients that have abundant antibody production [33–35]. A previous serological study of leprosy patients enrolled in U-MDT/CT-BR showed that the qualitative ML Flow test at baseline had limited sensitivity and specificity to predict whether patients would develop RR or ENL during follow-up [30]. The ML flow test uses PGL-I antigen and the ELISA anti PGL-I results from the current study did not show predictive value for ENL confirming previous results. The new finding of anti LID-1 serology showing predictive value for ENL is probably related to the antigen employed in a quantitative assay. While, PGL-I is a phenoglicolipid that induces IgM antibodies, LID-1 is a di-fusion recombinant protein originated from two highly immunogenic *M*. *leprae* antigens (ML2331 and ML0405). In the current study, *M*. *leprae*-specific ELISA results confirmed the limitation observed with ML flow results to predict RR. This limitation probably reflects the fact that RR mainly occurs in a context of strong CMI with little or no impact on the antibody production.

Initially our analysis at diagnosis showed higher seropositivity and antibody levels to PGL-I, LID-1 and ND-O-LID antigens in patients that developed reactions during follow-up compared to the ones that remained reaction-free. Further stratification of leprosy serology data according to the type of leprosy reaction showed higher antibody levels to all three antigens in both ENL and RR *versus* reaction-free patients. A gradual increase in both seropositivy rate and median ODs was seen from reaction-free to RR patients while ENL patients had the highest values. However, for patients that remained reaction-free throughout the follow-up, a variable rate (2–15%) had high serologic responses which were considered outliers. Therefore, our results showed a significant overlap between serologic responses of patients that further developed RR and the ones that remained reaction-free a finding that emphasizes the limitation of serology as a predictor of RR.

The impact of patients´ bacillary load in leprosy-specific serology has been well described [14, 18, 24, 36]. Also, higher BI has been shown to be an important factor for the development of reactions [12, 37, 38].To understand the impact of patients´ BI in this different serologic pattern observed from reaction-free to ENL, serology data of reactional (RR, ENL) and reaction-free patients was analyzed according to distinct BI range. These analyses showed a positive correlation between the rate of reaction and the BI: the frequency of reactions was low among BI negative patients (10%; 23/219), increasing to 50% (36/72) in patients with intermediary BI (0<BI<3) reaching 66% in reactional patients with high BI (≥3). These results corroborate the influence of BI in the development of leprosy reactions, as previously described [12,37,38]. Corroborating this observation, a previous *U-MDT/CT-BR* report showed that patients with high BI (≥3) had a higher frequency of reactions compared to patients with BI <3 throughout the follow-up and that recurrent reaction were associated with high BI (≥3) [7]. It is well known that in patients with high bacillary load the start of leprosy treatment is characterized by a massive release of mycobacterial antigens [39, 40] which can stimulate an exacerbated immune response, including antibody production and trigger leprosy reactions.

ENL is a severe, often difficult to manage immunological complication of borderline lepromatous (BL) and lepromatous leprosy (LL) that can be triggered by specific treatment [33]. Over 50% of lepromatous leprosy patients and 25% of borderline lepromatous leprosy patients experienced ENL prior to MDT [34]. ENL is characterized by exacerbated humoral immune response with increased synthesis of IgG1 [52] and transient activation of cellular immunity, demonstrated by Th1 type cytokine production [34]. In our study increased levels of anti-LID-1 antibodies at diagnosis were observed in patients who developed ENL during monitoring compared with reaction-free patients. A previous study showed that the LID-1 fusion protein is recognized by *M*. *leprae* specific antibodies and induces cellular immunity measured by IFNγ production [22]. A recent study among MB patients that presented RR or ENL at diagnosis or during MDT showed that high and persistent levels of anti-LID-1 was associated with the occurrence of ENL at diagnosis or during MDT [53]. Similarly, as indicated by the high AUC by the ROC analysis, anti-LID-1 serology at diagnosis could identify patients susceptible to develop ENL with 71% sensitivity and 80% specificity. The demonstrated capacity of original proteins that compose LID-1 fusion protein (ML2331 and ML0405) to induce both *M*. *leprae* specific humoral and cellular immune responses in leprosy patients supports the highest accuracy of anti LID-1 serology for ENL prediction. As part of ENL, immune complex formation and deposition that occur in tissues may cause a decrease in circulating antibodies levels [54]. Serological analysis of sequential samples collected during monitoring of patients from *U-MDT/CT-BR* might clarify the dynamic of anti-LID-1 serology during ENL.

The immunopathogenesis of RR is characterized by Th1 type immunity and increased pro-inflammatory cytokines, as IP-10, IFNγ, IL-1, IL-2 and IL-12 [41]. The association of high levels of anti-PGL-I antibodies and higher risk to develop of RR is controversial. Some studies associated high levels of anti-PGL-I antibodies at diagnosis or after treatment and higher risk to RR development [42–44]. However, other investigations showed similar anti-PGL-I levels among patients that developed RR and reaction-free patients [45–47]. Although, our results indicated higher antibody responses in RR patients compared to reaction-free patients, 10–15% of the reaction-free patients with negative BI had high outlier antibody levels, however these levels were lower compared to BI positive patients. Therefore, despite the statistically significant difference in positivity rate and medians of OD in BI negatives that developed RR and reaction-free ones, an important overlap was observed in antibody levels between these two groups. These results highlight that regardless of the further development of leprosy reactions, there is a high inter-individual variability of serological responses in reaction-free, RR and ENL patients and this variability results in extensive standard deviation. Also, in the group of patients with higher BI (≥ 3) there was no difference in antibody responses between patients that manifested RR and patients that remained reaction-free. Overall, these results and the area under the curve given by the ROC analysis for RR strongly indicate that serological markers present a questionable applicability for RR prognosis.

The identification of laboratory markers to predict the occurrence of leprosy reactions remains a priority in leprosy research aiming to prevent irreversible sequelae. Several studies have been carried out aiming to find a diagnostic and prognostic biomarker for leprosy reactions. CXCL10 and IL-6 were shown to be potential plasma markers for the diagnosis of RR and IL-7, PDGF-BB and IL-6 for ENL diagnosis [48]. The elevation of CXCL10 levels was associated with episodes of RR, however without positive predictive value [49]. A recent study evaluated TNFα, anti-ceramide, anti-S100, IgG and IgM anti-PGL-I, IgG1 and anti-LAM IgG3 antibodies in leprosy patients before, during and after the reaction episode. This study showed that preceding the RR episode 47% patients had increased levels of markers and the association of two to four markers detected 70% of patients that developed RR. The markers that showed the highest elevation were anti-ceramide, TNFα, anti-PGL-I and anti-S100 antibodies, suggesting that the association of these markers may enhance the sensitivity to predict RR [50]. Recently, biomarker profiles associated with the onset of RR were described in cohorts of patients from Bangladesh, Brazil, Ethiopia and Nepal who had peripheral blood mononuclear cells (PBMCs) stimulated and anti PGL-I antibodies measured. High IFN-γ, IP-10, IL-17- and VEGF production by *M*. *leprae*-stimulated PBMC peaked at diagnosis of RR and the ratio of these pro-inflammatory cytokines versus IL-10 could be useful for the early diagnosis of RR and for evaluating treatment efficacy. Nevertheless, anti-PGL-I serology was not useful for the diagnosis of RR, but could help treatment monitoring [51]. Overall our results showed low applicability of anti-PGL-I serology for the prognosis of leprosy reactions. Thus, together with our previous ML flow study, our results indicate low applicability of serology for both diagnosis and prognosis of RR. Therefore, other plasma biomarkers associated with anti-PGL-I serology could potentially increase the sensitivity of anti PGL-I to predict RR.

Despite the existence of seropositive patients with high BI who did not develop reaction and seronegative patients with low BI that developed reactions, anti-LID-1 serology at diagnosis has shown prognostic value for ENL development in BI positive patients: 71% sensitivity and 80% specificity. These apparently paradoxal results indicate that besides the impact of the bacillary load on the immune responses and on the risk to develop reactions, other yet unidentified factors are probably implicated in the susceptibility to manifest leprosy reactions.

## Supporting information

S1 Checklist STROBE Statement form—Leprosy Reactions: The Predictive Value of *Mycobacterium leprae*-Specific Serology Evaluated in a Brazilian Cohort of Leprosy Patients (U-MDT/CT-BR). (DOC)

S1 Table Main characteristics of the 452 patients stratified according to the reactional status during follow-up. (DOC)

S2 Table Summary of Receiver Operating Curve (ROC) results (DOC)

S1 Fig Baseline serological profiles to PGL-I, LID-1 and ND-O-LID *M*. *leprae* antigens in patients stratified according to Ridley & Jopling groups: (A) ELISA IgM responses to PGL-I antigen; (B) IgG responses to LID-1 antigen; (C) IgM and IgG responses to ND-O-LID. Cut-offs PGL-I: OD>0.25; LID-1: OD>0.3; ND-O-LID: OD>0.923. The median OD value of each group is represented by the horizontal line within box. The numbers above each box represent the positivity rate and the points above each box correspond to outlier ODs. The numbers below each box represent the number of patients. OD: optical density; TT: tuberculoid; BT: borderline tuberculoid; BB: borderline; BL: borderline lepromatous; LL: lepromatous leprosy.
(TIFF)

S2 Fig The time elapsed between blood intake and the development of reaction in the first year. Cut-offs PGL-I: OD>0.25; LID-1: OD>0.3; ND-O-LID: OD>0.923. The median OD value of each group is represented by the horizontal line within box.
(TIFF)

S3 Fig Correlation of BI and antibody levels to PGL-I (A, B, C), LID-1 (D, E, F) and ND-0-LID (G, H, I) among reactional (ENL and RR) and reaction-free patients. Each point represents the response of a single individual.
(TIF)
