# Supplementary material for: Leprosy reactions: The predictive value of Mycobacterium leprae-specific serology evaluated in a Brazilian cohort of leprosy patients (U-MDT/CT-BR)
Source: PLoS Negl Trop Dis. 2017 Feb 21;11(2):e0005396. doi: 10.1371/journal.pntd.0005396 (PMC5336302; doi:10.1371/journal.pntd.0005396)
Supplement: S1 Checklist — (DOC) [file pntd.0005396.s001.doc]

***Leprosy Reactions: The Predictive Value of Mycobacterium leprae-Specific Serology Evaluated in a Brazilian Cohort of Leprosy Patients (U-MDT/CT-BR)***

STROBE Statement form

|  | | Item No | Recommendation | | Lines |
| --- | --- | --- | --- | --- | --- |
| **Title and abstract** | | 1 | (*a*) Indicate the study’s design with a commonly used term in the title or the abstract | | ------ |
|  |  |  | (*b*) Provide in the abstract an informative and balanced summary of what was done and what was found | | 23-52 |
| Introduction | | | | |  |
| Background/rationale | | 2 | | Explain the scientific background and rationale for the investigation being reported | 126-135 |
| Objectives | | 3 | | State specific objectives, including any prespecified hypotheses | 129-132 |
| Methods | | | | |  |
| Study design | | 4 | Present key elements of study design early in the paper | | 126-135 |
| Setting | | 5 | Describe the setting, locations, and relevant dates, including periods of recruitment, exposure, follow-up, and data collection | | 138-149 |
| Participants | | 6 | (*a*) Give the eligibility criteria, and the sources and methods of case ascertainment and control selection. Give the rationale for the choice of cases and controls | | 150-156 |
|  |  |  | (*b*) For matched studies, give matching criteria and the number of controls per case | | Not Applicable |
| Variables | | 7 | Clearly define all outcomes, exposures, predictors, potential confounders, and effect modifiers. Give diagnostic criteria, if applicable | | 142-149  150-156  167-172 |
| Data sources/ measurement | | 8* | For each variable of interest, give sources of data and details of methods of assessment (measurement). Describe comparability of assessment methods if there is more than one group | | 138-142  173-206 |
| Bias | | 9 | Describe any efforts to address potential sources of bias | | 150-152 |
| Study size | | 10 | Explain how the study size was arrived at | | 153-156 |
| Quantitative variables | | 11 | Explain how quantitative variables were handled in the analyses. If applicable, describe which groupings were chosen and why | | 208-217 |
| Statistical methods | | 12 | (*a*) Describe all statistical methods, including those used to control for confounding | | 218-223 |
|  |  |  | (*b*) Describe any methods used to examine subgroups and interactions | | Not Applicable |
|  |  |  | (*c*) Explain how missing data were addressed | | Not Applicable |
|  |  |  | (*d*) If applicable, explain how matching of cases and controls was addressed | | ---- |
|  |  |  | (*e*) Describe any sensitivity analyses | | 221 |
| Results | | | | |  |
| Participants | | 13* | | (a) Report numbers of individuals at each stage of study—eg numbers potentially eligible, examined for eligibility, confirmed eligible, included in the study, completing follow-up, and analysed | ------- |
|  |  |  |  | (b) Give reasons for non-participation at each stage | ------- |
|  |  |  |  | (c) Consider use of a flow diagram | ------- |
| Descriptive data | | 14* | | (a) Give characteristics of study participants (eg demographic, clinical, social) and information on exposures and potential confounders | 157-166 |
|  |  |  |  | (b) Indicate number of participants with missing data for each variable of interest | Not Applicable |
| Outcome data | | 15* | | Report numbers in each exposure category, or summary measures of exposure | Not Applicable |
| Main results | | 16 | | (*a*) Give unadjusted estimates and, if applicable, confounder-adjusted estimates and their precision (eg, 95% confidence interval). Make clear which confounders were adjusted for and why they were included | Not Applicable |
|  |  |  |  | (*b*) Report category boundaries when continuous variables were categorized | 318-333, 342-354, 363-375 |
|  |  |  |  | (*c*) If relevant, consider translating estimates of relative risk into absolute risk for a meaningful time period | -------- |
| Other analyses | 17 | Report other analyses done—eg analyses of subgroups and interactions, and sensitivity analyses | | | 208-217 |
| Discussion | | | | |  |
| Key results | 18 | Summarise key results with reference to study objectives | | | 267-293 |
| Limitations | 19 | Discuss limitations of the study, taking into account sources of potential bias or imprecision. Discuss both direction and magnitude of any potential bias | | | 257-265 |
| Interpretation | 20 | Give a cautious overall interpretation of results considering objectives, limitations, multiplicity of analyses, results from similar studies, and other relevant evidence | | | 411-521 |
| Generalisability | 21 | Discuss the generalisability (external validity) of the study results | | | 522-528 |
| Other information | | | | |  |
| Funding | 22 | Give the source of funding and the role of the funders for the present study and, if applicable, for the original study on which the present article is based | | | At Plos NTD form |

*Give information separately for cases and controls.
