# Supplementary material for: Leprosy reactions: The predictive value of Mycobacterium leprae-specific serology evaluated in a Brazilian cohort of leprosy patients (U-MDT/CT-BR)
Source: PLoS Negl Trop Dis. 2017 Feb 21;11(2):e0005396. doi: 10.1371/journal.pntd.0005396 (PMC5336302; doi:10.1371/journal.pntd.0005396)
Supplement: S1 Table — (DOC) [file pntd.0005396.s002.doc]

**S1 Table. Main characteristics of the 452 patients stratified according to the reactional status during follow-up.**

|  | **Gender**  (male/female) | **Age (years)**  median (range) | **R & J**  Classification | **BI**  median (range) |
| --- | --- | --- | --- | --- |
| **Reactional (n=160)** | 115/45 | 41 (6-65) | 2 TT, 28 BT, 3 BB, 99 BL, 28 LL | 3.5 (0-6) |
| RR (n=119) | 88/31 | 43 (6-65) | 2 TT, 28 BT, 3 BB, 86 BL | 3 (0-6) |
| ENL (n=41) | 27/14 | 35 (8-62) | 13 BL, 28 LL | 4.5 (3-5.6) |
| **Reaction-free (n=292)** | 157/135 | 41 (8-65) | 33 TT, 176 BT, 9 BB, 39 BL, 35 LL | 0 (0-6) |

**Legend Table 1** – RR: Reversal reaction; ENL: Erythema nodosum leprosum; R&J: Ridley & Jopling classification; TT: tuberculoid; BT: borderline tuberculoid; BB: borderline; BL: borderline lepromatous; LL: lepromatous leprosy; BI: bacilloscopic index.
