# Supplementary material for: Leprosy reactions: The predictive value of Mycobacterium leprae-specific serology evaluated in a Brazilian cohort of leprosy patients (U-MDT/CT-BR)
Source: PLoS Negl Trop Dis. 2017 Feb 21;11(2):e0005396. doi: 10.1371/journal.pntd.0005396 (PMC5336302; doi:10.1371/journal.pntd.0005396)
Supplement: S2 Table — (DOC) [file pntd.0005396.s003.doc]

**S2 Table. Summary of Receiver Operating Curve (ROC) results**

|  | **Specificity** | **Sensitivity** | **AUC** |
| --- | --- | --- | --- |
| **RR** |  |  |  |
| PGL-I | 80% (95% CI: 75-84%) | 44% (95% CI: 35-54%) | 0.702 |
| LID-1 | 80% (95% CI 75-84%) | 37% (95% CI 29-47%) | 0.688 |
| ND-O-LID | 80% (95% CI 75-84%) | 35% (95% CI 26-44%) | 0.668 |
| **ENL** |  |  |  |
| PGL-I | 80% (95% CI: 75-84%) | 58% (95% CI: 42-73%) | 0.761 |
| LID-1 | 80% (95% CI: 75-84%) | 71% (95% CI: 55-84%) | 0.847 |
| ND-O-LID | 80% (95% CI: 75-84%) | 58% (95% CI: 42-73%) | 0.799 |
