# Supplementary figures and images for: Leprosy reactions: The predictive value of Mycobacterium leprae-specific serology evaluated in a Brazilian cohort of leprosy patients (U-MDT/CT-BR)
Source: PLoS Negl Trop Dis. 2017 Feb 21;11(2):e0005396. doi: 10.1371/journal.pntd.0005396 (PMC5336302; doi:10.1371/journal.pntd.0005396)

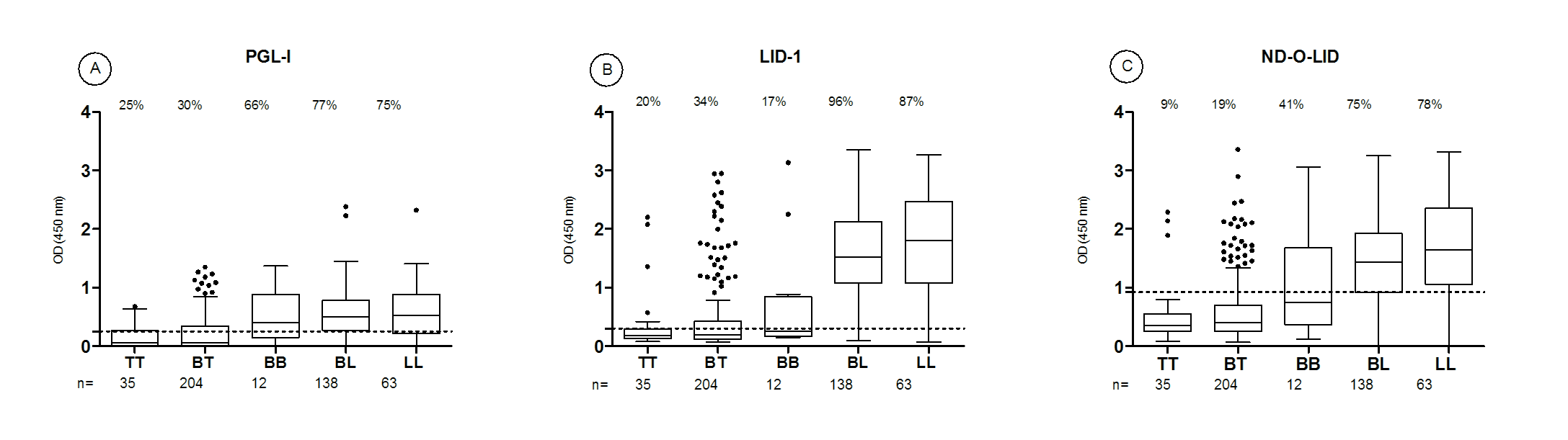

Supplement: S1 Fig — Baseline serological profiles to PGL-I, LID-1 and ND-O-LID M. leprae antigens in patients stratified according to Ridley & Jopling groups: (A) ELISA IgM responses to PGL-I antigen; (B) IgG responses to LID-1 antigen; (C) IgM and IgG responses to ND-O-LID. Cut-offs PGL-I: OD>0.25; LID-1: OD>0.3; ND-O-LID: OD>0.923. The median OD value of each group is represented by the horizontal line within box. The numbers above each box represent the positivity rate and the points above each box correspond to outlier ODs. The numbers below each box represent the number of patients. OD: optical density; TT: tuberculoid; BT: borderline tuberculoid; BB: borderline; BL: borderline lepromatous; LL: lepromatous leprosy. (TIFF) [file pntd.0005396.s004.tiff]

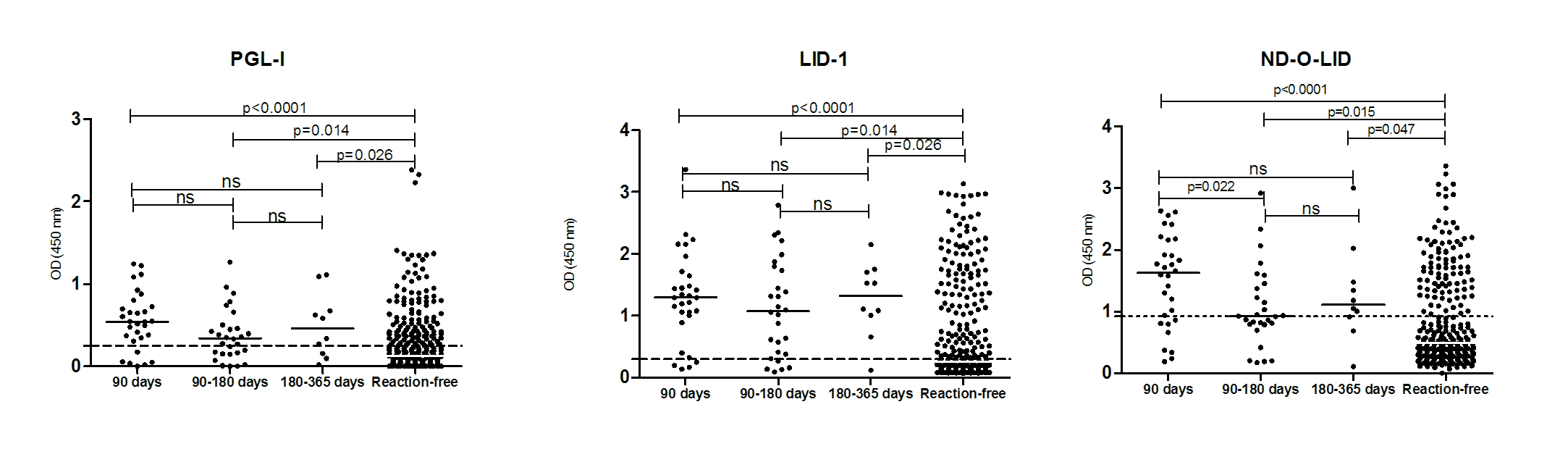

Supplement: S2 Fig — Cut-offs PGL-I: OD>0.25; LID-1: OD>0.3; ND-O-LID: OD>0.923. The median OD value of each group is represented by the horizontal line within box. (TIFF) [file pntd.0005396.s005.tiff]

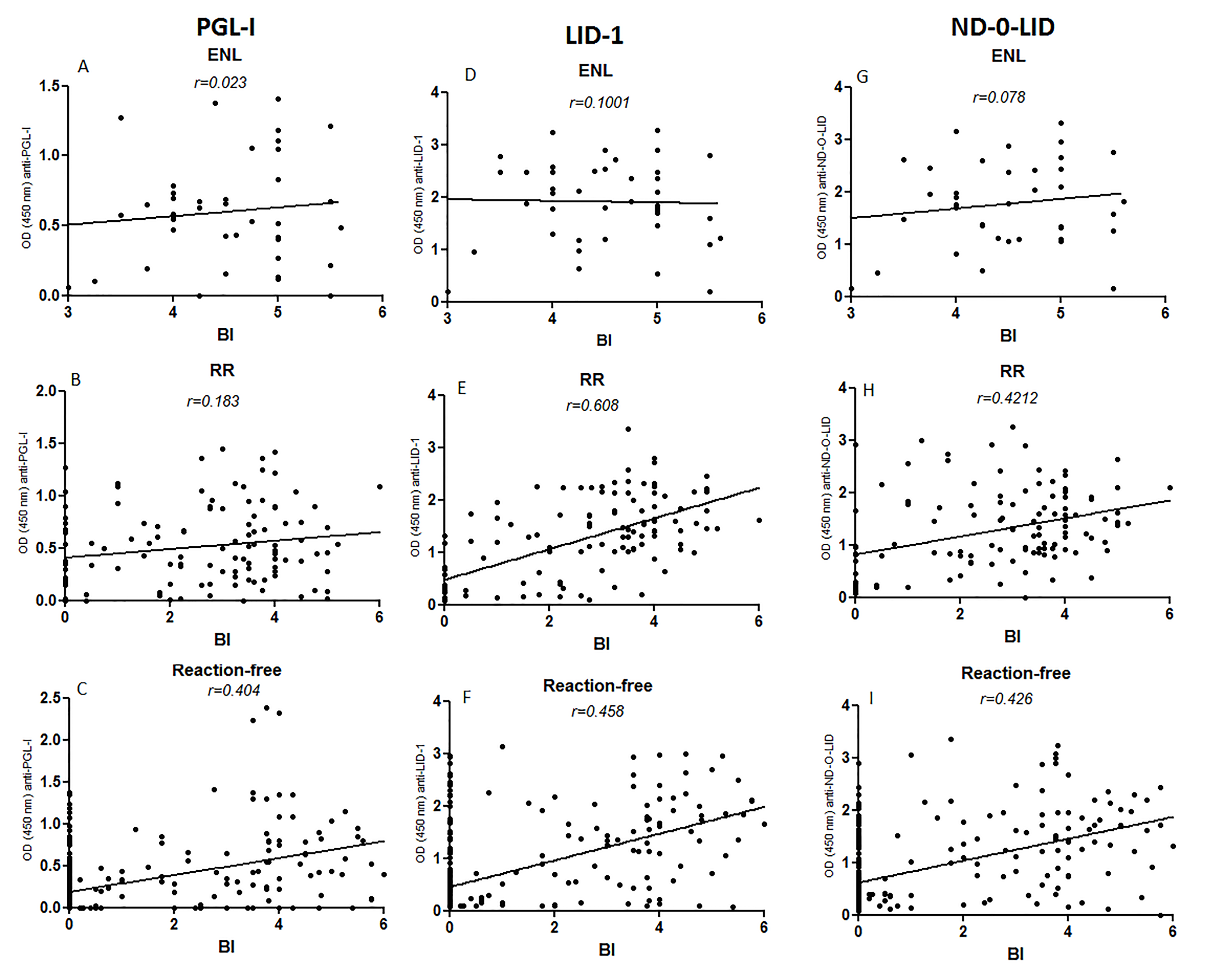

Supplement: S3 Fig — Correlation of BI and antibody levels to PGL-I (A, B, C), LID-1 (D, E, F) and ND-0-LID (G, H, I) among reactional (ENL and RR) and reaction-free patients. Each point represents the response of a single individual. (TIF) [file pntd.0005396.s006.tif]
